# The Novel Transcription Factor BnaA01.KAN3 Is Involved in the Regulation of Anthocyanin Accumulation Under Phosphorus Starvation

**DOI:** 10.3390/plants14132036

**Published:** 2025-07-03

**Authors:** Li He, Shan Peng, Ruihua Lin, Jiahui Zhang, Peng Cui, Yi Gan, Hongbo Liu

**Affiliations:** College of Advanced Agricultural Sciences, Zhejiang A & F University, Hangzhou 311300, China; 19851335721@163.com (L.H.); 15565691285@163.com (S.P.); lrh_0207@163.com (R.L.); z1277236091@163.com (J.Z.); cuipeng626@163.com (P.C.)

**Keywords:** *Brassica napus*, transcription factor, expression pattern, phosphorus starvation, biological function

## Abstract

The investigation of phosphorus metabolism and regulatory mechanisms is conducive to maintaining stable production of crops within a low-phosphorus environment. In phosphorus signal transduction, a few phosphorus starvation response (PHR) transcription factors were identified to bind to the characteristic *cis*-element, namely the PHR1 binding sequence (P1BS). While the molecular function of the PHR transcription factor has been intensively elucidated, here, we explore a novel transcription factor, BnaA01.KAN3, that undergoes specific binding to the P1BS by yeast one-hybrid and electrophoretic mobility shift assays, and its expression is induced with low-phosphorus stress. BnaA01.KAN3 possessed transcriptional activation and was located in the nucleus. The spatiotemporal expression pattern of *BnaA01.KAN3* exhibited tissue specificity in developmental seed, and its expression level was especially high 25–30 days after pollination. Regarding the phenotype analysis, the independent heterologous overexpression lines of *BnaA01.KAN3* in *Arabidopsis thaliana* exhibited not only significantly longer taproots but also an increased number of lateral roots compared to that of the wild type undergoing low-phosphorus treatment, while no differences were seen under normal phosphorus conditions. Furthermore, these lines showed higher anthocyanin and inorganic phosphorus contents with normal and low-phosphorus treatment, suggesting that *BnaA01.KAN3* could enhance phosphorus uptake or remobilization to cope with low-phosphorus stress. In summary, this study characterized the transcription factor *BnaA01.KAN3* that modulates low-phosphate adaptation and seed development, providing insights for improving phosphorus use efficiency and yield traits in *Brassica napus*.

## 1. Introduction

Phosphorus is an indispensable element for crop growth and development. As a non-renewable resource, phosphorus has increasingly attracted attention in terms of its efficient utilization for agricultural production security [1]. Although only 0.1–0.5% of dry matter in crops contains phosphorus, organic forms of phosphorus such as nucleic acids and phospholipids account for up to 85%, directly participating in key physiological processes such as energy metabolism, photosynthetic production, and cell proliferation [2,3]. As a major component of cell membranes, phospholipids are crucial for maintaining membrane structural stability and regulating cellular functions [4,5]. In response to abiotic stress, fatty acid derivatives enhance cellular osmotic adjustment capacity and inhibit membrane lipid peroxidation, forming a unique feedback regulation mechanism. They also alter metabolic pathways and restructure membrane lipids to improve the physiological utilization of phosphorus, effectively enhancing the stress resistance of crops [6,7]. On the other hand, resistance mechanisms optimize root architecture (such as root hair proliferation) and establish mycorrhizal symbiotic systems to enhance phosphorus acquisition capabilities [8]. Moreover, phosphorus signaling is involved in regulating stress response pathways, enhancing crop resistance by mediating hormone metabolism [9].

KANADI is a gene family unique to higher plants. All members of this family contain a conserved GRAP domain and have transcriptional activation activity. There are four members of the KANADI gene family in *A. thaliana*, namely KAN1-4. Previous studies have shown that KAN1, KAN2, and KAN3 participate in the polarity establishment of leaves [10]. The double mutant *kan1*/*kan2* and triple mutant *kan1*/*kan2*/*kan3* show significant polarity defects in leaves, with the adaxial side of the leaves forming protruding structures, and the floral organs also show obvious adaxialization characteristics. Additionally, the double mutant *kan1*/*kan2* also undergoes the phenomenon of early flowering, which indicates that it enters the reproductive stage earlier and that the transition from the juvenile to adult stage is advanced [11].

Rapeseed (*B. napus* L.) is an important vegetable oil, accounting for approximately 15% of the world’s commodity. The Yangtze River region with phosphorus deficiency is the main production area of semi-winter *B. napus* in China. Rapeseed is extremely sensitive to phosphorus deficiency, and its growth and development as well as yield are significantly affected by the input of phosphorus fertilizer [12]. Under low phosphorus stress, the phenotype demonstrates slow growth; dark green or even purplish red leaves appear in the seedling stage; root development is delayed, leading to plant dwarfism; and the vegetative growth stage is prolonged, causing the flowering period to be delayed [13]. When the deficiency intensifies, the accumulation rate of the aboveground biomass decreases by more than 40%, the number of effective branches reduces by 30% to 50%, and the differentiation of pods is hindered, resulting in a sharp reduction in the number of pods per plant. This low-phosphorus state not only reduces the rapeseed yield by 20% to 60% but also weakens the stability of cell membranes, significantly reducing the survival rate of plants under low-temperature and drought stress [14,15]. In crops, the phosphorus metabolism network deeply affects the growth and development of crops and their stress response capabilities by regulating core processes such as lipid synthesis and signal transduction [16]. Therefore, there is an urgent need to explore the mechanism of the efficient utilization of phosphate fertilizer and select and breed low-phosphorus-tolerant varieties to ensure the stable yield of rapeseed, improve the utilization efficiency of phosphate fertilizer, and reduce agricultural source pollution.

## 2. Results

### 2.1. Transcription Factor BnaA01.KAN3 Binds to Cis-Element P1BS

**Figure 1:**
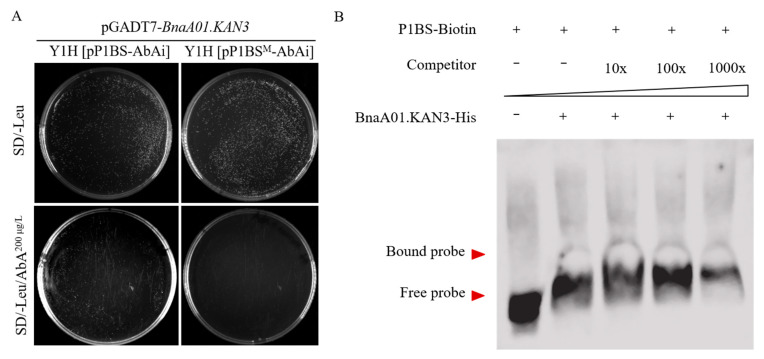
Identification of interaction between transcription factor BnaA01.KAN3 and *cis*-element P1BS by yeast one-hybrid assay (**A**) and EMSA (**B**). A: Yeast one-hybrid assay was used to confirm that transcription factor BnaA01.KAN3 interacted with *cis*-element P1BS in *in vivo*; B: EMSA was used to confirm in vitro interaction. BnaA01.KAN3-His: vector of pET28a contained *BnaA01.KAN3* gene.

The *cis*-element P1BS and its mutant were integrated into the pAbAi vector though *Hin*dIII and *Xho*I. Then, two types of vectors (pP1BS-AbAi and pP1BS^Mutant^-AbAi), linearized with *Bst*BI, were transformed into Y1HGold to construct bait yeast strains through homologous recombination into the genome of Y1HGold. The plasmid pGADT7-*BnaA01.KAN3* was transformed into two types of yeast strains, Y1H[pP1BS-AbAi] and Y1H[pP1BS^Mutant^-AbAi], to confirm the interaction. The yeast strain Y1H[pP1BS-AbAi] could grow on the SD/-Leu plus 200 μg/L AbA resistant medium but not Y1H[pP1BS^Mutant^-AbAi] (Figure 1A). Moreover, in the EMSA (electrophoretic mobility shift assay), the transcription factor BnaA01.KAN3 could bind to the *cis*-element P1BS, and even the hybridization signals became weakened with the increase in mutation competitor (Figure 1B). These results indicate that the transcription factor BnaA01.KAN3 can bind to the phosphorus starvation response element P1BS in *in vivo* and *in vitro*.

### 2.2. Homology and Phylogenetic Tree Analysis

**Figure 2:**
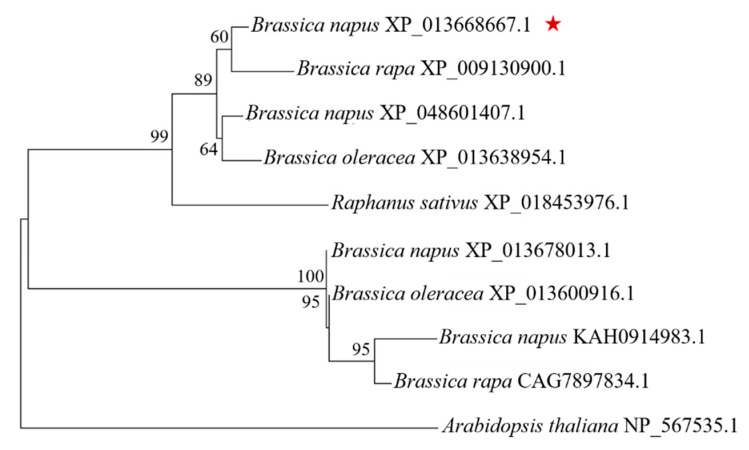
Phylogenetic tree of BnaA01.KAN3 with homologous protein sequence. Red star indicates *BnaA01.KAN3*. Homologous genes from other four species (*B. rapa B. oleracea*, *R. sativus*, and *A. thaliana*) were used in phylogenetic tree analysis.

There are four homologous *BnaKAN3s* in the ‘Zhongshuang 11’ variety of *B. napus*. The total length of the *BnaA01.KAN3* sequence is 939 bp, encoding 312 amino acids, with a molecular weight of 36.07 kDa. The conserved domain SHAQKYF class in the SANT superfamily was found in the protein sequence. A phylogenetic tree analysis revealed that the sequence of XP_013668667.1 from *Brassica rapa* had the closest genetic relationship to *BnaA01.KAN3*, and the other homologous genes are arranged from nearest to furthest as follows: *Brassica oleracea*, *Raphanus sativus*, and *A. thaliana* (Figure 2).

### 2.3. Tissue-Specific Expression Pattern and Response to Phosphorus Starvation

**Figure 3:**
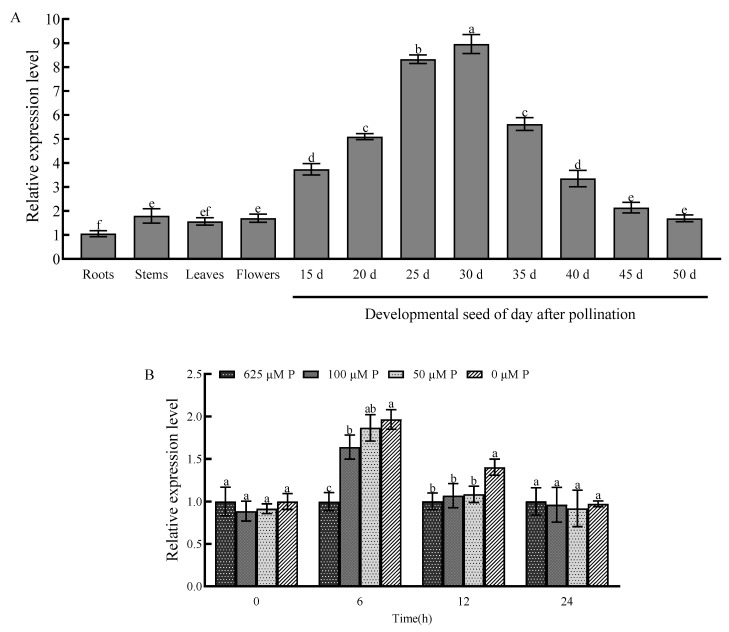
The relative expression level of *BnaA01.KAN3* in different tissues (**A**) and the response of *B. napus* to low-phosphorus stress (**B**). Values are shown as the means of three biological replicates, with error bars representing the standard deviation, and those with the same letter are not significantly different at *p* < 0.05. The statistical analysis showed the least significant difference.

The expression pattern of *BnaA01.KAN3* was analyzed by RT-qPCR in the roots, stems, leaves, flowers, and seeds in different developmental stages (15, 20, 25, 30, 35, 40, 45, and 50 days after pollination). The results show that the *BnaA01.KAN3* gene’s relative expression level was mainly detected in developmental seeds, especially 25 and 30 days after pollination, reaching 8.33 and 8.96 times higher than that of the roots, respectively (Figure 3A). This indicates that the molecular function is related to seed development, oil accumulation, and fatty acid component regulation. To further clarify the phosphorus starvation response pattern of *BnaA01.KAN3*, one normal phosphorus concentration (625 μM P) and three low phosphorus concentrations (100 μM P, 50 μM P, and 0 μM P) were applied to seedling for 0 h, 6 h, and 12 h. After that, the relative expression levels at different phosphorus concentrations were analyzed. It was found that the *BnaA01.KAN3* gene’s relative expression level was significant induced at all low phosphorus concentrations after 6 h of treatment compared to that at 0 h (Figure 3B). These results indicate that the *BnaA01.KAN3* gene can respond to phosphorus starvation and may be involved in the regulation of phosphorus deficiency in *B. napus*.

### 2.4. Transcription Auto-Activation of pGBKT7-BnaA01.KAN3

**Figure 4:**
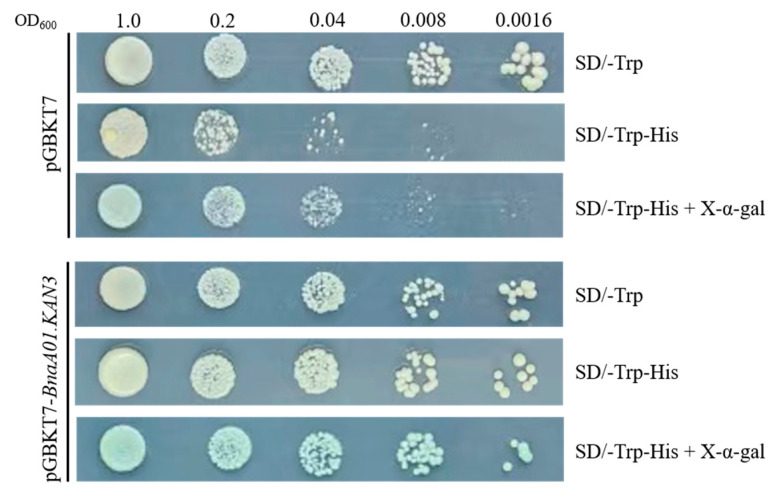
Transcriptional auto-activation analysis of BnaA01.KAN3.

The *BnaA01.KAN3* gene was cloned into yeast expression vector pGBKT7-BD by homologous recombination. The yeast strain Y2HGold that contained the recombinant vector pGBKT7-*BnaA01.KAN3* could grow on the SD/-Trp-His and SD/-Trp-His plus X-α-gal medium, activate *MEL-1* reporter genes, and encode α-galactosidase, making the yeast turn blue, which means that the transcription factor BnaA01.KAN3 activated the reporter genes (Figure 4). As a myb-like DNA-binding domain transcription factor, the transcriptional activation of BnaA01.KAN3 was confirmed by a yeast two-hybrid auto-activation system.

### 2.5. Subcellular Localization of Fusion Protein BnaA01.KAN3-GFP

**Figure 5:**
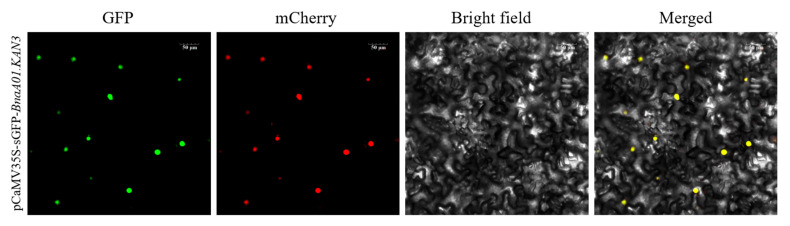
The subcellular localization of BnaA01.KAN3. The fluorescence signal of the fusion protein was observed in the epidermal cells of tobacco leaves. The OsD53-mCherry protein was located in the nucleus as a control.

To determine the subcellular localization of the transcription factor BnaA01.KAN3, a recombination plasmid of pCAMBIA1305.1-GFP-*BnaA01.KAN3* was constructed. Then, the BnaA01.KAN3-GFP fusion protein was transiently expressed in *Nicotiana tabacum* leaves with a OsD53-mCherry protein as a control. Compared to the nucleus location signal in OsD53-mCherry protein, the result shows that the green fluorescence signal of the BnaA01.KAN3-GFP fusion protein was located in the nucleus in the merged image (Figure 5).

### 2.6. Constructed Transformants with BnaA01.KAN3 Overexpression in A. thaliana

The heterologous overexpression vector p1305.1-35S-*BnaA01.KAN3*-Nos was constructed and transformed into the *A. thaliana* through *Agrobacterium tumefaciens* GV3101. The positive plants were identified by hygromycin and PCR (Supplementary Figure S1). For T_2_ generation seeds, 13 independent lines were confirmed to be single-copy insertion transgenic lines using Mendel’s law of inheritance segregation test (Supplementary Table S2). T_3_ generation seeds were harvested, and five independent lines were chosen for further functional analysis. In addition, the relative expression level of *BnaA01.KAN3* in five independent lines was significantly higher than that of the wild type (Supplementary Figure S2).

We have only analyzed the *BnaA01.KAN3* gene’s relative expression level using RT-qPCR in five independent Arabidopsis lines.

### 2.7. Phenotype Analysis

**Figure 6:**
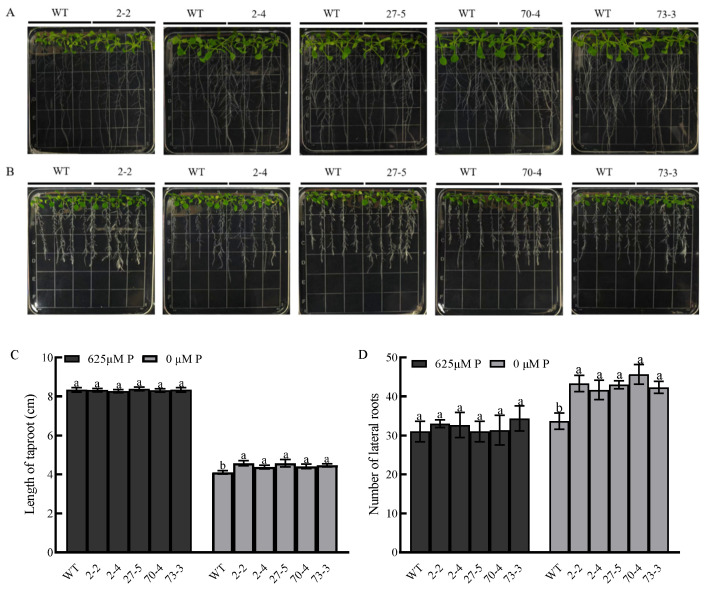
Seedling phenotypes and root statistics of independent lines with *BnaA01.KAN3* overexpression in *A. thaliana* under normal (625 μM P) and low-phosphorus treatments (0 μM P). (**A**) The above-ground and root phenotypes of the plantlet with normal phosphorus treatment. (**B**) The above-ground and root phenotypes of the plantlet with low-phosphorus treatment. (**C**) The length of the taproot between the wild type and overexpression lines with normal and low-phosphorus treatments. (**D**) The number of lateral roots between the wild type and overexpression lines with normal and low-phosphorus treatments. The values are shown as the means of three biological replicates, with error bars representing the standard deviation. Bars with the same letter are not significantly different at *p* < 0.05. The statistical analysis showed the least significant difference.

The wild type and five independent lines were obtained using normal and low-phosphorus treatments. After 7 days of treatment, we observed that there was no significant difference in the above-ground and roots phenotypes between the wild type and overexpression lines in the normal phosphorus medium (Figure 6A,C,D). However, under phosphorus starvation, five independent signal-copy insertion lines showed that the length of the taproots are significantly longer than that of the wild type (Figure 6B,C). Meanwhile, the same result was observed in the lateral root number between the overexpression lines and wild type (Figure 6B,D). An increase in the root specific surface area could improve the absorption of phosphorus.

### 2.8. Anthocyanin and Inorganic Phosphorus Measurement

**Figure 7:**
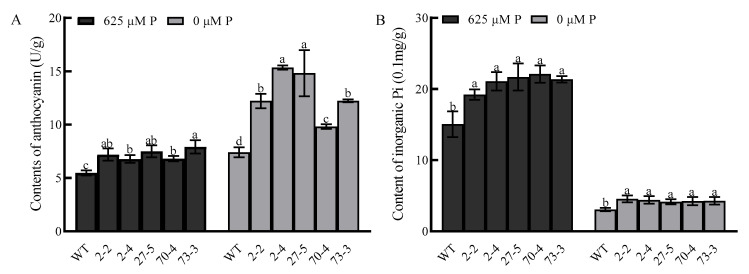
Anthocyanin (**A**) and inorganic phosphorus (**B**) analysis in wild type and overexpression lines under normal (625 μM P) and low-phosphorus treatments (0 μM P). Values are shown as means of three biological replicates, with error bars representing standard deviation; bars followed by same letter are not significantly different at *p* < 0.05. Statistical analysis showed least significant difference.

All *BnaA01.KAN3* overexpression lines showed a higher content of anthocyanin than the wild type under normal and low-phosphorus treatments, and even the increased proportion is higher in the overexpression lines (Figure 7A). Additionally, the overexpression lines possessed more inorganic phosphorus than the wild type when treated with normal and low-phosphorus treatments, but the decreased proportion is also higher in the overexpression lines (Figure 7B). These results imply that *BnaA01.KAN3* overexpression could enhance the tolerance to low-phosphorus stress in *A. thaliana*.

## 3. Discussion

The response to low-phosphorus stress is strictly regulated by a group of transcription factors in plants. As is well known, the P1BS was a key *cis*-element, serving as a conserved binding site by transcription factors in the signal transduction of low-phosphorus stress [17,18,19]. In this study, a novel KANADI family protein, BnaA01.KAN3, was observed to undergo specific binding to P1BS in *in vivo* and *in vitro*, respectively. Broadly, phosphorus starvation response gene expression does not particularly change under low-phosphorus treatment, which function as long-distance or systemic sensing pathways that trigger each other in plants [17]. According to the results of the *BnaA01.KAN3* gene’s relative expression level with the low-phosphorus treatment, we speculated that the transcription factor BnaA01.KAN3 is an important executor leading to precise regulation in the target gene. In addition, the tissue-specific expression pattern indicates that the *BnaA01.KAN3* gene is involved in phosphorus metabolism and recycling in seed development.

In allotetraploid *B. napus*, four homologous genes were presented in the A and C genomes. A phylogenetic analysis showed that *BnaA01.KAN3* had the highest homology to the gene from *Brassica rapa*, indicating that these genes have conserved evolution among cruciferous species. The specific molecular function of *BnaA01.KAN3* was characterized through heterologous overexpression in *A. thaliana*. The root morphology is the main phenotypic characteristic in plants adapting to low-phosphorus stress [20,21]. There were no significant differences in root length and the number of lateral roots between the wild type and overexpression lines cultured on the medium with a normal phosphorus concentration. However, the root length was longer and the number of lateral roots was higher than that of the wild type cultured on the medium with a low phosphorus concentration, respectively. On the other hand, a common physiological feature of the low-phosphorus response in plants is the development of anthocyanin accumulation brought about by anthocyanin biosynthetic enzymes [22]. The significant increase in anthocyanin may indicate that the *BnaA01.KAN3* gene could enhance the resistance of the overexpression lines under phosphorus-deficient conditions. Meanwhile, overexpression lines have more inorganic phosphorus than the wild type whether they are cultured on a medium with a normal or low phosphorus concentration. A possible explanation is that phosphorus absorption or cyclic metabolic utilization was enhanced by the *BnaA01.KAN3* gene’s overexpression [23,24].

## 4. Materials and Methods

### 4.1. Plant Materials

The *B. napus* cv. ‘Zhongshuang 11’, *N. tabacum*, and *A. thaliana* (*col-0*) were used as plant materials for gene cloning and genetic transformation, respectively. For plantlet greenhouse cultivation, 16/8 h light and dark cycles were carried out, with a 25 °C average temperature and 50–60% relative humidity.

### 4.2. Yeast One-Hybrid Assay and EMSA

The coding sequences of *BnaA01.KAN3* were cloned from the cDNA of ‘Zhongshuang 11’ through RT-PCR; the primers are listed in Supplementary Table S1. Then, the fragment was subcloned into the vectors pGADT7 and pET28a for the yeast one-hybrid assay and EMSA according to the protocols of the Matchmaker Gold Yeast One-Hybrid Library Screening System (Cat. No. 630491, Clontech, Mountain View, CA, USA) and the LightShift Chemiluminescent EMSA Kit (Cat. No. 20148, Thermo, Rockford, IL, USA). The *cis*-element P1BS and its mutant sequence were synthesized by two antiparallel oligonucleotides with overhanging sticky ends for cloning into the appropriately digested pAbAi vector. In addition, the probe was labeled by using the Biotin 3′ End DNA Labeling Kit (Cat. No. 89818, Thermo, Rockford, IL, USA).

### 4.3. Bioinformatics Analysis

Information about the *BnaA01.KAN3* gene was retrieved from the NCBI database, and species closely related to *B. napus* were selected for homologous analysis. A phylogenetic tree with nine homologous members of other Brassicaceae was created using MEGA software (version 7.0) with the neighbor-joining method, and the bootstrap test was repeated 1000 times.

### 4.4. RT-qPCR

For the tissue-specific expression pattern of *BnaA01.KAN3*, the total RNA was extracted from the roots, stems, leaves, flowers, and seeds in different developmental stages (15, 20, 25, 30, 35, 40, 45, and 50 days after pollination) of the ‘Zhongshuang 11’ cultivar. On the other hand, the total RNA from the plantlet with or without low-phosphorus treatment was used to analyze the response to phosphorus starvation. The *B. napus* SUMO E2 ligase encoding gene (*BnaUBC9*) was used as a reference gene. All of the primers are listed in Supplementary Table S1. The RT-qPCR was repeated three biological times, and the results were calculated according to the 2^−ΔΔCt^ analysis method [25]. The means of the relative expression level were analyzed for variance using the least significant difference test at the *p* < 0.05 level of significance with SPSS software (version 27.0.1).

### 4.5. Transcription Auto-Activation Analysis

In the transcription auto-activation assay, we cloned the *BnaA01.KAN3* gene through homologous recombination with the pGBKT7-FP and pGBKT7-RP sequences, which are listed in Supplementary Table S1. Then, the pGBKT7-BD empty vector and pGBKT7-BD-*BnaA01.KAN3* vector were transformed into the yeast strain Y2HGold with two reporter genes (*HIS3* and *MEL1*) and then spread on SD/-Trp, SD/-Trp-His and SD/-Trp-His plus X-α-gal plates, respectively. The plates were cultured at 30 °C in an incubator for 3–5 days to observe the growth of the yeast.

### 4.6. Subcellular Localization Analysis

The recombinant pCAMBIA1305.1-35S-*BnaA01.KAN3*-GFP vector was transformed into *A. tumefaciens* GV3101 for transient expression in tobacco leaves. Mixed bacterial infection liquid including OsD53-mCherry as a nuclear marker was injected into the epidermis of tobacco leaves and dark-cultured for 48 h. Fluorescence signal was detected using an LSM980 laser scanning confocal microscope (Carl Zeiss, Oberkochen, Germany). For GFP signals, 488 nm and 505–530 nm were used as the excitation and emission wavelengths, respectively, while 587 nm and 600–630 nm wavelengths were used for OsD53-mCherry signals.

### 4.7. Plant Transformation and Confirmation

For *BnaA01.KAN3* overexpression, the pCAMBIA1305.1-35S-Nos recombinant vector, by homologous recombination, was used for transformation into *A. thaliana* (*col-0*) through mediated *A. tumefaciens* GV3101. The positive transformants were identified by PCR. For screened single-copy insertion transgenic lines, the seeds of independent T_2_ were detected following Mendel’s law of inheritance segregation through the chi-square test. The formula is χ^2^ = |A − 3 × B|^2/[3 × (A + B)], where A and B represent the numbers of positive and negative plants, respectively. If χ^2^ is less than 3.84, this indicates that the transgenic line is a single-copy insertion at the significant level of 0.05. Then, 10 plants from each candidate line were transplanted to the soil and normally cultivated in the greenhouse until T_3_ generation seeds were harvested. Furthermore, the non-separated T_3_ lines were selected for the low-phosphorus treatment analysis.

### 4.8. Phenotype and Physiological Indicator Analysis with Low-Phosphorus Treatment

The wild type (*col-0*) and five independent lines (No. 2-2, 2-4, 27-5, 70-4, and 73-3) of single-copy insertion homozygous T_3_ seeds were cultured in a greenhouse as previously described. For a phenotype analysis of tap and lateral roots, 7 days after germination, the seedlings were transferred onto 1/2 MS solid medium with normal (625 μM P) and low-phosphorus (0 μM P) treatment for 7 days. Meanwhile, the contents of anthocyanin and inorganic phosphorus were measured in the wild type and overexpression lines with or without treatment, respectively. After seed germination, the seedlings were transferred to 1/5 Hoagland liquid nutrient solution for 3 weeks. Then, the treatment was conducted as previously mentioned. The determination of anthocyanin and inorganic phosphorus contents was conducted according to the protocols of the Test Kit (ADS116P0 and ADS099TC1, AIDISHENG). All experiments were repeated three biological times. The means of the data were analyzed for variance using the least significant difference test at the *p* < 0.05 level of significance using SPSS software.

## 5. Conclusions

The transcription factor BnaA01.KAN3, which is located in the nucleus and has transcriptional activation, is a key factor in the response to low phosphorus. The above results indicate that the transcription factor BnaA01.KAN3 could promote anthocyanin accumulation and inorganic phosphorus absorption through binding to the low-phosphorus response *cis*-element P1BS. Furthermore, in *A. thaliana* overexpression lines, *BnaA01.KAN3* significantly reshapes the root architecture, increasing the length of the taproot and enhancing the formation of lateral roots. Thus, this work will provide insights for improving phosphorus use efficiency in the abiotic breeding of *B. napus*.

## Data Availability

The original contributions presented in this study are included in the article and Supplementary Materials; further inquiries can be directed to the corresponding author.

## Supplementary Materials

The following supporting information can be downloaded at: https://www.mdpi.com/article/10.3390/plants14132036/s1, Table S1: Information of primer sequences; Table S2: The segregation ratio of overexpression independent T_2_ lines; Figure S1: PCR identification of T_1_ lines; Figure S2: The relative expression level of *BnaA01.KAN3* in 5 independent single copy insertion T_3_ lines.
