# Supplementary material for: The Novel Transcription Factor BnaA01.KAN3 Is Involved in the Regulation of Anthocyanin Accumulation Under Phosphorus Starvation
Source: Plants (Basel). 2025 Jul 3;14(13):2036. doi: 10.3390/plants14132036 (PMC12251713; doi:10.3390/plants14132036)
Supplement: Supplementary file 1 [file plants-14-02036-s001.zip › plants-3706430-supplementary.pdf]

Supplementary Table S1 Information of primer sequences

Primers	Sequence 5'-3'	Length (bp)	Application
P1BS-FP	AGCTTTTGCATATACGTTTGCATATACGTTTGCATATACGTC	42	Yeast one-hybrid
P1BS-RP	TCGAGACGTATATGCAAACGTATATGCAAACGTATATGCAAA		
P1BS^{Mutant}-FP	AGCTTTTAAACGGTTGTTTAAACGGTTGTTTAAACGGTTGTC	42	Yeast one-hybrid
P1BS^{Mutant}-FP	TCGAGACAACCGTTTAAACAACCGTTTAAACAACCGTTTAAA		
pET28a-FP	TGTCGACGGAGCTCGAATTCGGGAGGAAGGTTTGGTATAG	978	EMSA
pET28a-RP	ACAGCAAATGGGTCGCGGATCCATGGAGCTTTTCCCTTCA		
EMSA-FP	AGACTTTGATTTGCATTATTGCATATACGTAGTAGTATAATGGTACTATC	50	EMSA
EMSA-RP	GATAGTACCATTATACTACTACGTATATGCAATAATGCAAATCAAAGTCT		
BnaA01.KAN3-FP	CATCTACAGATGTATCGGACCATC	158	qRT-PCR
BnaA01.KAN3-RP	CGAGCTTCACTTGAGGAGTTAGTC		
pGBKT7-FP	ATATGGCCATGGAGGCCGAATTCATGGAGCTTTTCCCTTCACAAC	985	Transcription auto-activation

pGBKT7-RP	CCGCTGCAGGTCGACGGATCCCTAGGGAGGAAGGTTTGGTAT		
35S-GFP-FP	GAGGACAGCCCAGATCAACTAGTATGGAGCTTTTCCCTTCACA	988	Subcellular localization
35S-GFP-RP	TCCTCGCCCTTGCTCACCATGGATCCCTAGGGAGGAAGGTT		
p1305.1-35S-FP	GCCACCATGGATGGAGCTTTTCCCTTCACAACCT	959	Overexpression
p1305.1-35S-RP	GCCAACGCGTCTAGGGAGGAAGGTTTGGTATAGC		
PCR-FP	GAAGCTCGGTTCCACTTAAA	591	Transformants identification
PCR-RP	GAACGATCGGGGAAATTCGAGC		
BnaUBC9-FP	GCATCTGCCTCGACATCTTGA	68	Reference gene
BnaUBC9-RP	CGATAGCAGCACCTTGGAGATA		

Note: the restriction enzyme sites are underlined. FP: Forward primer, RP: Reverse primer.

Supplementary Table S2 The segregation ratio of overexpression independent T_2 lines

T_2 independent lines	Positive	Negative	Total plants	χ^2 value
2	203	58	261	1.07
16	122	42	166	0.03
25	125	40	165	0.05
27	116	33	149	0.65
44	93	25	118	0.92
45	124	35	159	0.76
49	112	41	156	0.26
54	109	31	140	0.61
67	95	28	123	0.33
70	122	30	162	2.25
71	199	57	256	1.02
73	109	30	139	0.87
78	94	25	119	1.01

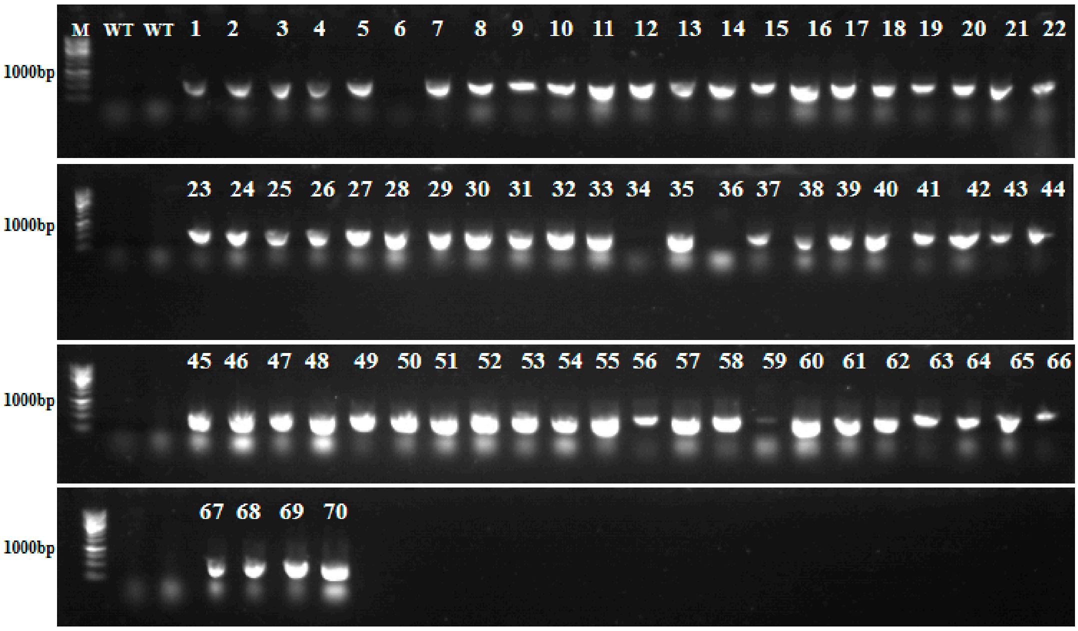

Supplementary Figure S1 PCR identification of T_1 lines.

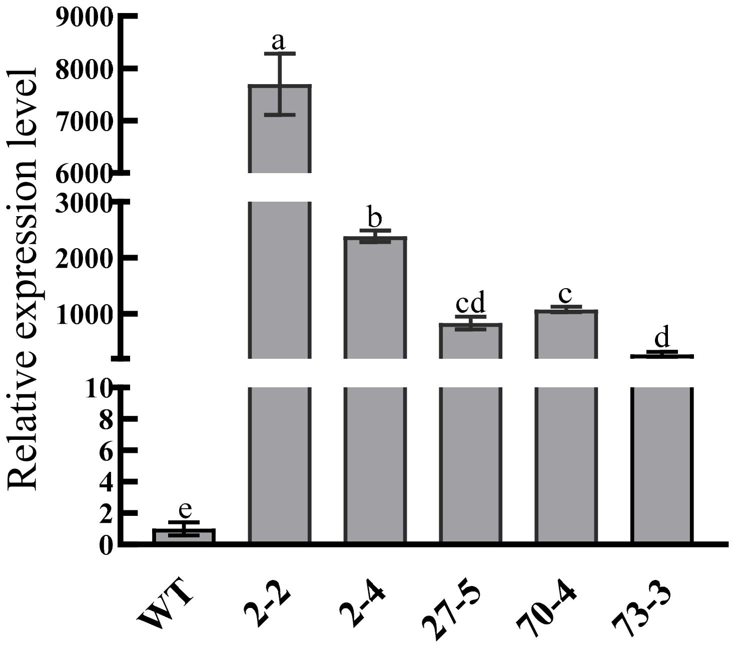

Supplementary Figure S2 The relative expression level of *BnaA01.KAN3* in 5 independent single copy insertion T_3 lines.
